# Concurrent monoclonal gammopathy and systemic lupus erythematosus in a known case of ulcerative colitis: A case report

**DOI:** 10.1002/ccr3.5063

**Published:** 2021-11-09

**Authors:** Mahan Shafie, Alireza Hadizadeh, Soheil Khalaji, Samaneh Parsa

**Affiliations:** ^1^ Tehran University of Medical Sciences Tehran Iran; ^2^ NeuroTRACT Association Students' Scientific Research Center Tehran University of Medical Sciences Tehran Iran; ^3^ School of Medicine Tehran University of Medical Sciences Tehran Iran; ^4^ Tehran University of Medical Sciences Tehran Iran; ^5^ Department of Internal Medicine Imam Khomeini Hospital Complex Tehran Iran

**Keywords:** monoclonal gammopathy, systemic lupus erythematosus, Ulcerative colitis

## Abstract

Our patient had previously been diagnosed with Ulcerative colitis. The clinical manifestations of the patient along with laboratory tests such as anti‐dsDNA and proteinuria were also positive. Therefore, the clinical manifestation was consistent with SLE. In the following work up, monoclonal gammopathy in serum electrophoresis was also detected.

## Graphical abstract

The association between UC and SLE is a rare incidence. We expect polyclonal hypergammaglobulinemia in serum protein analysis in patients with SLE; however, the results showed monoclonal gammopathy. This observation added even more peculiarity.

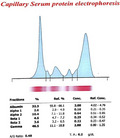

## 1 BACKGROUND

Many autoimmune diseases have been reported to be associated with Ulcerative Colitis (UC). The association between UC and systemic lupus erythematosus (SLE) has been rarely reported in some case reports, however, none of them consider a relationship or a link between UC and SLE. This may be due to SLE colitis may mimic UC and some drugs for UC treatment, particularly sulphasalazine, may cause drug‐induced SLE.^1^, ^2^ In addition, the serum protein electrophoresis of the patients with SLE usually shows polyclonal gamma globulin rises due to hyperactivity of B‐Lymphocytes and monoclonal gammopathy is a rare finding in serum electrophoresis in a few cases.^1^, ^3^ In this case report, we reported a patient with a past medical history of UC and diagnosed with SLE and revealed monoclonal gammopathy in serum electrophoresis. To the extent of our knowledge, this is the first case of a UC that has been diagnosed with SLE and monoclonal gammopathy simultaneously.

## 2 CASE PRESENTATION

**FIGURE 1:**
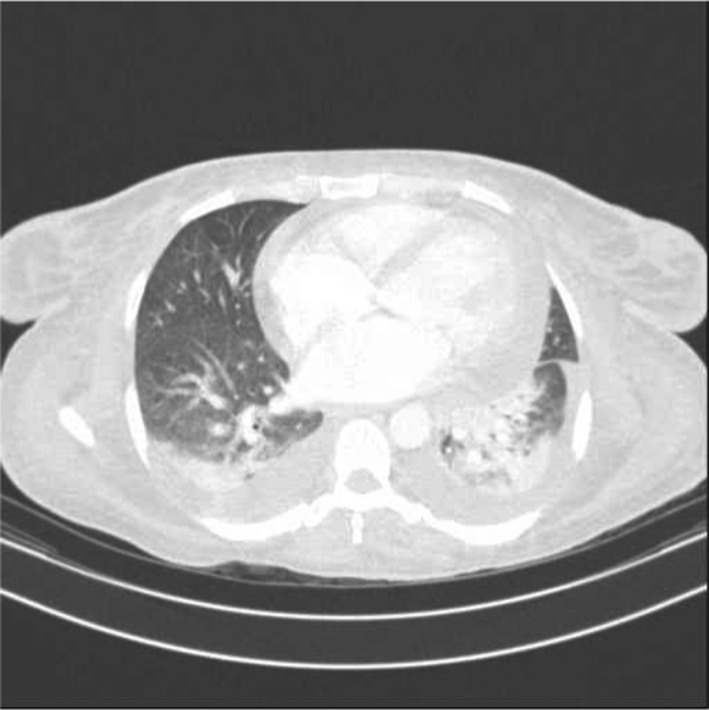
Axial thoracic CT scan cut of lower thoracic region set at pulmonary window; this cut depicts pleural effusion, ground‐glass opacity and collapse consolidation

**FIGURE 2:**
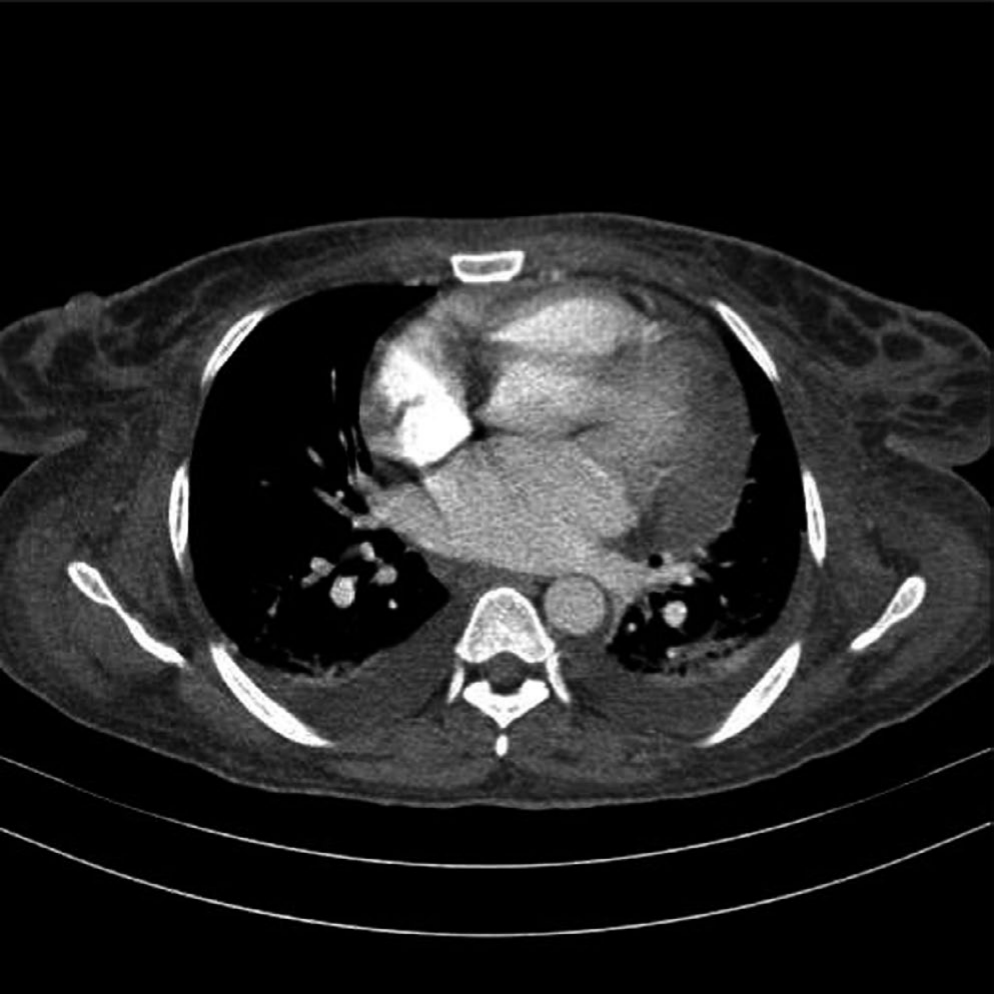
Axial thoracic CT scan cut of lower thoracic region set at thoracic window; in this cut pericardial effusion is also noticeable

**FIGURE 3:**
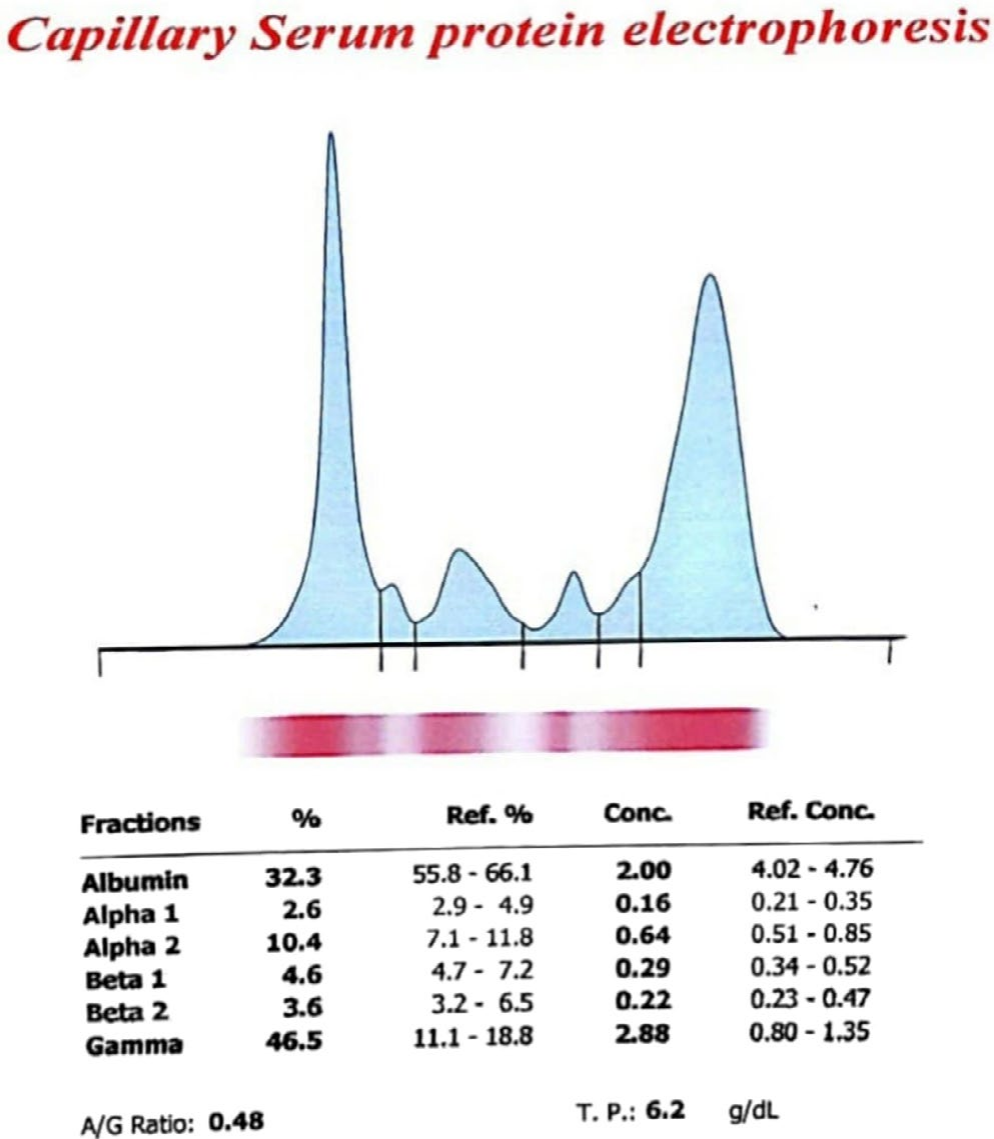
Capillary serum protein electrophoresis

A 38‐year‐old woman complaining of bloody diarrhea and general fatigue was admitted to the emergency department and was then hospitalized. She stated that she was being treated for Ulcerative colitis for the past 9 months and her symptoms had started a year ago with generalized fatigue and weight loss. After 3 months she presented other symptoms including abdominal pain and rectal bleeding therefore, upper endoscopy and colonoscopy was carried out. When she was referred to our center, she had been under treatment for 3 months and her medical record which included upper gastrointestinal endoscopy and colonoscopy revealed crypt destructive colitis and chronic gastritis. With these findings, she was diagnosed with early UC and was under treatment with mesalazine (3 g daily) and pantoprazole (40 mg daily). In addition, her routine blood tests had revealed pancytopenia before. This had led the doctor to obtain a bone marrow biopsy which was inconclusive due to an unsatisfactory sample, and also to request rheumatologic laboratory tests which were reported negative (Figures 1, 2, 3).

The patient showed symptoms of general fatigue and lethargy along with fever and night sweats. She also stated that she had involuntarily lost 14 kg in 3 months. Her family members also stated that their younger sibling also had a history of an unknown rheumatologic disease and was expired years ago. Her physical examinations showed bilateral pitting edema in both legs, oral ulcers. She also suffered from bloody diarrhea and hemoptysis. When she was admitted she was severely ill. Her lower limb forces were reduced (3/5) and she could barely stand or walk. Her neck examination showed enlarged lymph nodes on the back of her neck. However, her vital signs were stable. As the patient's clinical signs were suggestive for SLE or myositis we ordered prednisolone (1 mg/kg) before the laboratory results were ready. This significantly improved limb forces and hemoptysis.

Her laboratory tests were as shown in the table (Tables 1, 2). The significant findings were pancytopenia, mildly elevated liver enzymes, a high ESR, severely low reticulocyte count, high LDH and B12 levels. Her urine analysis revealed hematuria and bacteriuria. Her arterial blood gases were normal. As her hemoglobin levels were deemed too low, she received 1 unit of packed RBC. We also obtained another bone marrow biopsy which was not in favor of malignancies. Another notable and important finding is that her serum protein electrophoresis revealed an unusual abnormality which was increased gamma globulin levels while albumin levels were low (Table 3). This presentation was suggestive of gammopathy.

**TABLE 1:** Patient's lab data on admission

ANA	5.6
p‐ANCA	Negative
c‐ANCA	Negative
RF	Negative
Anti Ro	Negative
Anti La	Negative
Anti‐dsDNA	>300
Lupus Anticoagulant	5.6
GBM‐Ab	Negative
Anti‐centromere Ab	Negative
Anti‐SCL70 Ab	Negative
Wright	Negative
Coombs Wright	Negative
2ME	Negative
PPD	Negative
24h Urine	Volume = 1600 ml
	Protein = 496 mmol/24 h
	Cr = 400 mmol/24 h
	Ca = 3 mmol/24 h

Abbreviations: 2ME, 2‐Mercaptoethanol; ANA, anti‐nuclear antibody; anti‐centromere Ab, *anti*‐*centromere antibodies*; Anti‐dsDNA, *anti*‐*double stranded DNA*; Anti‐SCL70 Ab, anti‐topoisomerase I; Ca, Calcium; c‐ANCA, cytoplasmic anti‐neutrophil cytoplasmic antibodies; Cr, Creatinine; *GBM*‐*Ab*, anti–glomerular basement membrane; p‐ANCA, perinuclear anti‐neutrophil cytoplasmic antibodies; PPD, purified protein derivative; RF, rheumatoid factor.

**TABLE 2:** Patient's lab data during hospitalization

	Day 1	Day 2	Day 3	Day 4	Day 5	Day 6	Day 7	Day 8	Day 9	Day 10	Day 11
WBC (count)	2100	1500	1400	1700	1500	1700	2400	1900	3100	4000	4500
Hb (%)	7.9	8.2	7.9	7.2	9.6	8.5	10.2	9.2	8.9	9.9	10.1
Plt (count)	100,000	127,000	103,000	84,000	97,000	93,000	85,000	107,000	123,000	132,000	139,000
Urea (mg/dl)	37	27	16	16	17	26	27	27	20	30	31
Cr (mg/dl)	1.3	1.2	1.1	1	0.9	0.9	0.9	0.9	0.9	0.9	0.9

Abbreviations: Cr, Serum Creatinine; Hb, Hemoglobin; Plt, Platelet; WBC, white blood cell.

**TABLE 3:** Capillary serum protein electrophoresis

Fractions	%	Ref. %	Conc.	Ref. Conc.
Albumin	32.3	55.8–66.1	2.00	4.02–4.76
Alpha−1	2.6	2.9–4.9	0.16	0.21–0.35
Alpha−2	10.4	7.1–11.8	0.64	0.51–0.85
Beta−1	4.6	4.7–7.2	0.29	0.34–0.52
Beta−2	3.6	3.2–6.5	0.22	0.23–0.47
Gamma	46.5	11.1–18.8	2.88	0.80–1.35

Note

A/G Ratio = 0.48; Total Protein = 6.2 g/dl.

In her following work up a spiral chest computed tomography (CT) scan without contrast was requested that revealed ground‐glass opacities in the lower lobes of both lungs. This finding was more prominent in the left lung. This finding was also suggestive of pneumonia. Other remarkable findings were mosaic attenuation in the parenchyma of both lungs, air cyst (12 ×12 mm) in left upper lobe, bilateral mild pleural effusion, cardiomegaly, and mild pericardial effusion.

As the findings were compatible with pneumonia, antibiotic therapy was initiated and she received 4.5 g of piperacillin/tazobactam intravenously 3 times a day and 750 mg of levofloxacin daily orally.

Due to swelling and edema in her lower limbs, she underwent venous color Doppler sonography and nothing remarkable was observed. The patient also underwent neck sonography and many reactive lymph nodes were found on both sides of the neck. The largest ones were (30 ×9.5 mm) in zone 2 of the right side and the other (23 ×7 mm) in zone 2 of the left side. Other sonographic evaluations revealed axillary lymph nodes as big as (31 ×15 mm) on the left and (25 ×17 mm) on the right, respectively.

She also underwent echocardiography to assess cardiac activity and the prominent finding was a decreased ejection fraction (EF) (45%). As her medical condition and the hematochezia had worsened 5mg of prednisolone 3 times a day and also a single unit of IVIG 60 mg was ordered.

As the clinical manifestations of the patient were consistent with SLE, a rheumatologic consultation was requested. Which suggested laboratory tests that turned out to be positive for anti‐dsDNA and also her urine analysis manifested severe proteinuria. Meanwhile, she had already shown symptoms of polyserositis, mouth ulcers, and pancytopenia. With all the aforementioned findings and signs, SLE became a confirmed diagnosis for the patient. Thus, intensive therapy with rituximab induction therapy along with 500 mg of prednisolone pulse therapy for 3 days was started, we also used 200 mg of hydroxychloroquine daily simultaneously with a daily dose of 1.5 g of mycophenolic acid as a maintenance therapy.

Unfortunately, though, 2 days after the initiation of therapy oxygen saturation levels suddenly dropped to 70% and the patient became dyspneic thus the patient was intubated. Unfortunately, the patient showed signs of hemoptysis which was suggestive of alveolar hemorrhage. She was intubated and 2 units of fresh frozen plasma (FFP) were transfused. An emergent chest X‐RAY was obtained that showed diffused patchy infiltrates that resembled either pneumocystis pneumonia (PCP), alveolar hemorrhage, or pulmonary thromboembolism (PTE). Portable echocardiography showed an even more decreased EF (40%) along with mild right ventricle enlargement and moderate left ventricle dysfunction. thereafter a new course of antibiotic therapy started with intravenous (IV) cotrimoxazole, vancomycin, and cefepime. The patient received another 500 mg of methylprednisolone pulse therapy and 4 mg of dexamethasone IV, 3 times a day. However, the patient expired on the same day due to alveolar hemorrhage arrest.

## 3 DISCUSSION

There is no comprehensive consensus about the origin and etiology of UC however it does fulfill the criteria for an autoimmune disease.^4^, ^5^ Some of the characteristics of UC are precisely autoimmune. The fact that certain human leukocyte antigens (HLAs) are in association with UC,^5^ certain autoantibodies are found to be involved as well, and most importantly the response to immunosuppressive therapy has led to this conclusion.^4^, ^5^ Our patient had previously been diagnosed and was under treatment for the disease and her medical history was consistent with our findings as well. However, her early workup was unsuccessful in diagnosing the underlying cause for pancytopenia. This phenomenon is thought to be related to the early stages of SLE in which it had no clinical manifestation.^4^, ^6^

A wide variety of disorders are also thought to have an autoimmune basis such as celiac disease, ankylosing spondylitis, primary sclerosing cholangitis, etc. one of such disorders that is rarely concurrent with UC is SLE.^7^ In a case‐control study conducted by Shor et al. UC was more prevalent in patients diagnosed with SLE.^8^ However, in multivariate regression models, SLE was not in association with UC. There is a certain problem that also makes establishing the relation between the two even more difficult and that is the fact that certain medications, 5‐aminosalicylic acid (5‐ASA) compounds, in particular, can cause drug‐induced lupus.^2^ And sometimes it's hard to distinguish between these two. Even though lupus can be both idiopathic and drug‐induced, there are certain differences between the two. While anti‐dsDNA antibodies and more importantly hypocomplementemia are common findings among idiopathic cases and they are rarely associated with drug‐induced type.^2^ Even though our patient had been on 5‐ASA medication for a considerable amount of time we believe that her SLE diagnosis was true and accurate as she fulfilled the EULAR/ACR criteria and the time gap between initiation of therapy and manifestations indicates so.

In a study conducted by Snook Ja, the association between autoimmune diseases and inflammatory bowel disease had been studied and it turns out that 6.6% of the patients with UC had another autoimmune disease.^4^, ^9^ However, the gastrointestinal manifestations of both UC and SLE are similar and common in both, and might be difficult to distinguish between the two; particularly in lupus colitis which is caused by inflammation, vasculitis, and necrosis in vessels of the intestine. Kurlander and Kisner described the involvement in patients with concurrent SLE and UC to be mostly in 2 forms^10^; (1) a predominating vasculitis with bleeding and perforation usually associated with a clinical picture suggestive of SLE; or (2) non‐specific ulcerative or granulomatous colitis.

Although many cases have been suggestive for the association between UC and SLE, the link between the two needs more study and more investigation as to its too rare.

Another disease manifestation of our patient which was interesting was monoclonal gammopathy. This phenomenon is rarely associated with SLE however several mechanisms have been proposed to be involved; polyclonal B‐cell activation both peripheral and in the bone marrow, abnormal B‐cell clones, And immunosuppressive agents prescribed for SLE patients that disposes them to malignancies especially multiple myeloma, are some of these mechanisms.^1^, ^3^ In a study conducted by YM Ali, et al at the University of Toronto they concluded that the prevalence of monoclonal gammopathy in SLE patients (5.4%) is higher than the general population (1%–2%).^3^ In this study, it's also suggested that this disorder is not associated with any malignancies. Our patient had a very high ESR level this inflammatory factor had prompted us to obtain a serum protein electrophoresis. As the pathogenicity of gammopathy in such patients has not been discovered we suggest that all patients go through malignancy workup.

## 4 CONCLUSION

The association between UC and SLE seems to be a rare phenomenon and it is not fully clear yet whether it is due to a common physiopathology or not. On the other hand, we expected the serum protein analysis of our patient to be in favor of polyclonal hypergammaglobulinemia as she was diagnosed with SLE, however, the results showed monoclonal gammopathy. Epidemiological links between SLE and monoclonal gammopathy and to a lesser extent ulcerative colitis has been suggested by previous studies, but the unusual phenomenon we observed was the concurrency of all three in a single case.

## CONFLICT OF INTEREST

The authors have no conflict of interest to declare.

## AUTHOR CONTRIBUTIONS

MS contributed in developing the research idea and composing and revising the manuscript. AH contributed in composing and revising the manuscript. SK contributed in composing and revising the manuscript. SP contributed in developing the research idea and revising the manuscript.

## ETHICS APPROVAL

This study was approved by the research and ethics committee of Tehran University of Medical Sciences. The patient’s family have given their informed consent to publish this case.

## CONSENT

Written informed consent was obtained from the patient’s next of kin for publication of this case report and any accompanying images. A copy of the written consent is available for review by the Editor‐in‐Chief of this journal.

## Data Availability

Data sharing is not applicable to this article as no datasets were generated or analyzed during the current study.
